# Review of Electrohydraulic Actuators Inspired by the HASEL Actuator

**DOI:** 10.3390/biomimetics10030152

**Published:** 2025-03-02

**Authors:** Levi Tynan, Upul Gunawardana, Ranjith Liyanapathirana, Osura Perera, Daniele Esposito, Jessica Centracchio, Gaetano Gargiulo

**Affiliations:** 1 School of Engineering, Design and Built Environment, Western Sydney University, Kingswood, NSW 2747, Australia; 18409680@student.westernsydney.edu.au (L.T.); u.gunawardana@westernsydney.edu.au (U.G.); r.liyanapathirana@westernsydney.edu.au (R.L.); o.perera@westernsydney.edu.au (O.P.); 2 Department of Information and Electrical Engineering and Applied Mathematics, University of Salerno, Via Giovanni Paolo II, 132, 84084 Fisciano, Italy; daesposito@unisa.it; 3 Department of Electrical Engineering and Information Technologies, University of Naples Federico II, Via Claudio 21, 80125 Napoli, Italy; jessica.centracchio@unina.it

**Keywords:** soft robotics, electrohydraulic actuators, HASEL actuator, fluidic actuator, DEA, HAXEL actuator

## Abstract

The muscle-like movement and speed of the electrohydraulic actuator have granted it much attention in soft robotics. Our aim is to review the advancements in electrohydraulic actuators inspired by the Hydraulically Amplified Self-healing Electrostatic (HASEL) actuator. With this paper, we focus on the performance of 21 electrohydraulic actuator designs developed across five Universities, ranging from the earliest HASEL designs to the latest electrohydraulic designs. These actuators reported up to 60 N forces and contracting strains of up to 99%. The actuators with the best overall performance so far have been the Quadrant HASEL actuator and the HEXEL actuator, developed at the University of Colorado Boulder. However, notable is also the HALVE actuator (produced by ETH Zürich, Switzerland), which, by using a 5 µm PVDF-TrFE-CTFE film with a relative permittivity of 40, produced 100 times the electrostatic force of any of the electrohydraulic actuators under review. The latter shows that there is room for improvement as low force and displacement still limit the viability of the soft actuators in real-life applications.

## 1. Introduction

Soft robotics replaces many of the rigid materials used in traditional robots with more compliant materials to better mimic how nature creates movement [1]. These compliant materials create robots that are more adaptable to moving in rough terrain and handling more delicate tasks. There are many technologies emerging in the field of soft robotics. Unfortunately, all of them have their drawbacks [1,2,3]. Fluidic Actuators (FA) are the most common soft robotics technology due to their high force production. However, though the fluidic actuator itself is made of compliant materials, it needs to be controlled by external components that are rigid and bulky. Dielectric Elastomer Actuators (DEA), on the other hand, are a type of soft robotics that produce force locally and with a high energy density. However, it is difficult to scale up the force production, and they also require high voltages for control. Inspired by these two technologies, researchers at the University of Colorado Boulder developed the Hydraulically Amplified Self-Healing Electrostatic (HASEL) actuator [4,5].

**Figure 1:**
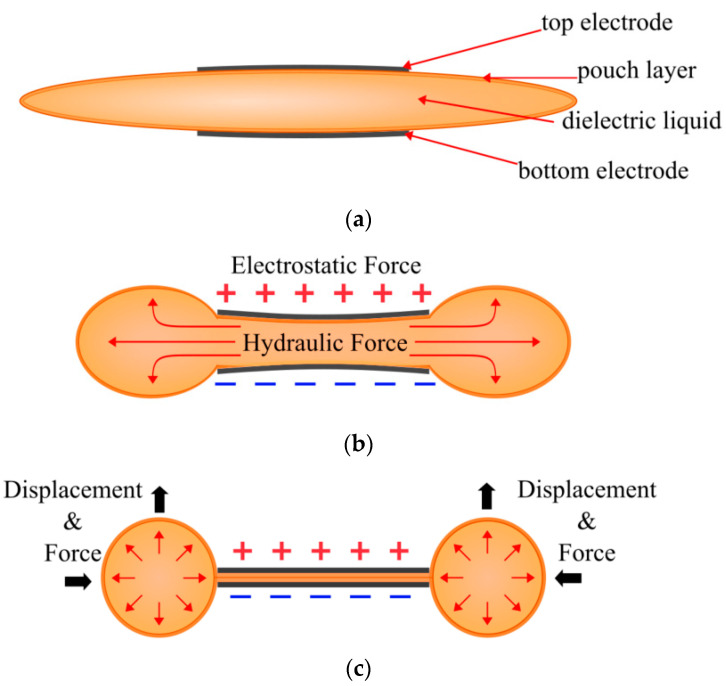
Depiction of the HASEL actuator: (**a**) when at rest, displaying the electrodes, the pouch layer, and dielectric liquid; (**b**) when a voltage is applied, producing electrostatic and hydraulic forces, where the red arrows indicate the hydraulic force and displacement inside the pouch; (**c**) when fully zipped, with the black arrows demonstrating the direction of force and displacement where a load can be attached.

The development of the HASEL actuator brought about a new field of soft robotics, more broadly referred to as electrohydraulic actuators. The first HASEL actuator consisted of an elastomer pouch with electrodes on both sides and filled with a dielectric fluid, depicted in Figure 1a. When a high voltage is applied, generally between 2 and 10 kV, an electrostatic force is produced between the electrodes, squeezing the pouch and displacing the fluid, as shown in Figure 1b. This displacement generates a hydraulic force that can be used to displace a load (Figure 1c).

Since the development of the HASEL actuator, a number of research groups have created their own electrohydraulic actuators. Almost all the electrohydraulic actuators developed by these researchers can be linked back to the elastomeric HASEL actuator and the Peano HASEL actuator, which will be discussed below. This paper provides a collection of these electrohydraulic actuators developed over the years, discussing their major addition to the field and their performance compared to the HASEL actuators. Dielectric materials were also compared and contrasted. The main metrics that will be highlighted will be force and strain, as electrohydraulic actuators are often lacking in these areas [6]. Obtaining specific metrics, such as specific power and specific energy, allows us to normalise the results on a per-kilogram basis for comparative analysis of performance.

## 2. Types of Electrohydraulic Actuators

### 2.1. Elastomeric HASEL Actuators

Many principles of the field were established with the Elastomeric HASEL actuators [4,7,8]. Elastomeric HASEL actuators operate as described in Figure 1. The main distinguishing feature of the elastomeric HASEL actuator is that the soft pouch is made with an elastomeric material. In this case, the elastomers included 0.5 mm Ecoflex 00-30 (Smooth-on) and 0.3 mm polydimethylsiloxane (PDMS). The core principle of soft elastomeric materials is that they bend and stretch when the dielectric fluid deforms the outer pouch. The dielectric fluid used was Envirotemp FR3. Stretchable parallel electrodes are also used to amplify this effect. The electrodes were made from Polyacrylamide (PAM) hydrogels that contain lithium chloride (LiCl).

**Figure 2:**
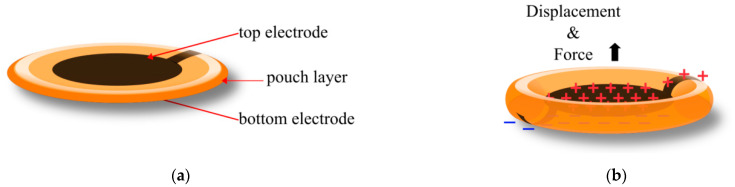
Elastomeric donut HASEL actuator: (**a**) when at rest, showing the electrodes and pouch layer; (**b**) when a voltage is applied, indicating the direction of the force and displacement commonly used.

Two types of elastomeric HASEL actuators were developed: the planar HASEL actuator and the donut HASEL actuator. Donut HASEL actuators mostly rely on the displacement of the dielectric fluid, bending the soft pouch and lifting an attached load (Figure 2). The Donut HASEL actuator, with a 21 kV applied voltage, was able to produce a maximum strain under no load of approximately 50% (this metric is commonly referred to as free strain). Applying a load to the actuator reduced the maximum strain until it returned to 0% with a 250 g load applied, or 2.453 N (this metric is commonly referred to as the blocking force). By decreasing the size of the stretchable electrode, the free strain was reduced to approximately 40%, and the blocking load increased to 400 g (3.924 N).

**Figure 3:**
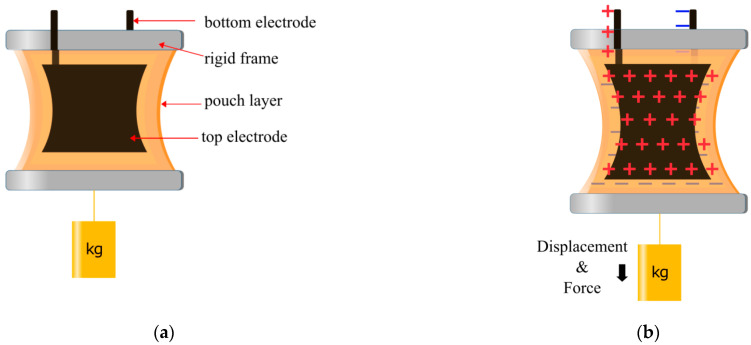
Planar HASEL actuator: (**a**) when at rest, displaying the electrode, pouch layer and the rigid frame; (**b**) when a voltage is applied, indicating the direction in which the force and displacement are commonly applied.

Planar HASEL actuators use the stretching of the actuator to displace a load. For optimal performance, the planar HASEL actuator requires a large rigid frame to stretch the elastomer on the transverse plane (Figure 3). This requirement, known as prestretching, reduces the adaptability of the actuator [5]. A single planar HASEL actuator produced 79% strain of a 250 g (2.452 N) load with approximately 22.5 kV applied voltage. Applying a sinusoidal 9 kV voltage to the actuator could produce a 16% strain of a 1.5 kg load (14.715 N). The peak-specific power and specific work of two planar HASEL actuators were reportedly 614 W/kg and 70 J/kg, respectively.

### 2.2. Peano HASEL Actuator (Thin Film Plastic Actuator)

**Figure 4:**
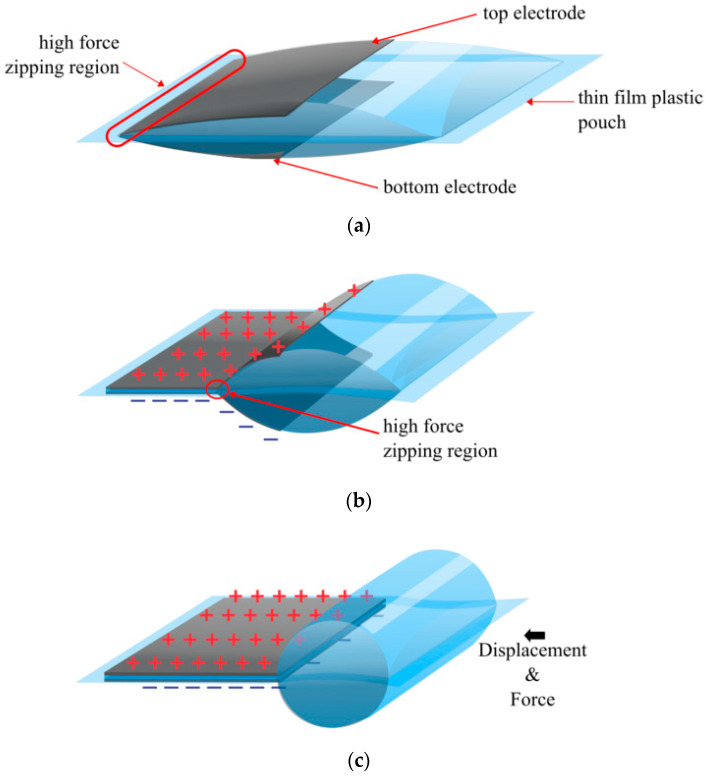
Peano HASEL actuator (**a**) with an initial applied voltage and electric field. (**b**) initial zipping from a high field region. (**c**) Fully zipped electrodes produce force and displacement.

Building on the success of the elastomeric actuators, a new type of actuator called the Peano HASEL actuator has been built, as shown in Figure 4 [5]. The most significant contribution of the Peano HASEL actuator is configuring the electrodes so they ‘zip’ together [9]. Unlike the elastomeric actuators, the electrodes are not parallel, as one side of the electrodes is placed very close together in what is called the ‘zipping region’ (Figure 4a). The zipping region creates a much larger electric field and squeezing (compressive) force at a much lower voltage. The high force of the zipping region pulls the rest of the electrodes into the high force region (Figure 4b,c). Peano HASEL actuators also benefit from replacing the soft elastomeric pouch with a non-stretchable thin film plastic material. Non-stretchable materials can transfer much more of the hydraulic force to pushing or pulling a load, whereas elastomers lose much of their force transfer with the stretching of the pouch. A Biaxially Oriented Polypropylene (BOPP) film with a thickness of 18–21 µm was selected as the thin film plastic material, as it could also be heat-sealed, making it easier to fabricate the pouch. The conductive layer used is generally made of carbon paint that can be painted or printed on with an inkjet printer [10]. The Peano HASEL actuator units were designed with three Peano HASEL actuators in series.

The performance of the Peano HASEL actuator has undergone many optimisations over the years. This was achieved through both calculations and simulations [11,12,13,14,15,16]. The Peano HASEL actuator unit has reportedly produced a maximum free strain of 15% and a maximum blocking force of 65 N. The peak specific power was 160 W/kg, and the average specific power was above 50 W/kg.

Attempts to overcome the low strain of the Peano HASEL actuator led to the development of the high-strain (HS) Peano HASEL actuator [14,17]. Much of the length of the Peano HASEL actuator was taken up by the electrode length, thereby reducing the maximum free strain possible. Electrodes were placed on the side of the pouch, making the HS Peano HASEL actuator [14]. The pouch was again made with 18 µm BOPP. However, Thermoplastic Polyurethane (TPU) film was also experimented with to reduce what was referred to as out-of-plane deformation with its elastomeric properties. The free strain of the HS Peano was reported to be ~24%; however, the blocking force was reduced to 18 N. The peak specific power, peak average power, and specific energy were reported as ~120 W/kg, ~78 W/kg, and 4.03 J/kg, respectively. The HS Peano HASEL actuator strain can be increased further in applications if a pulley system is implemented [14].

**Figure 5:**
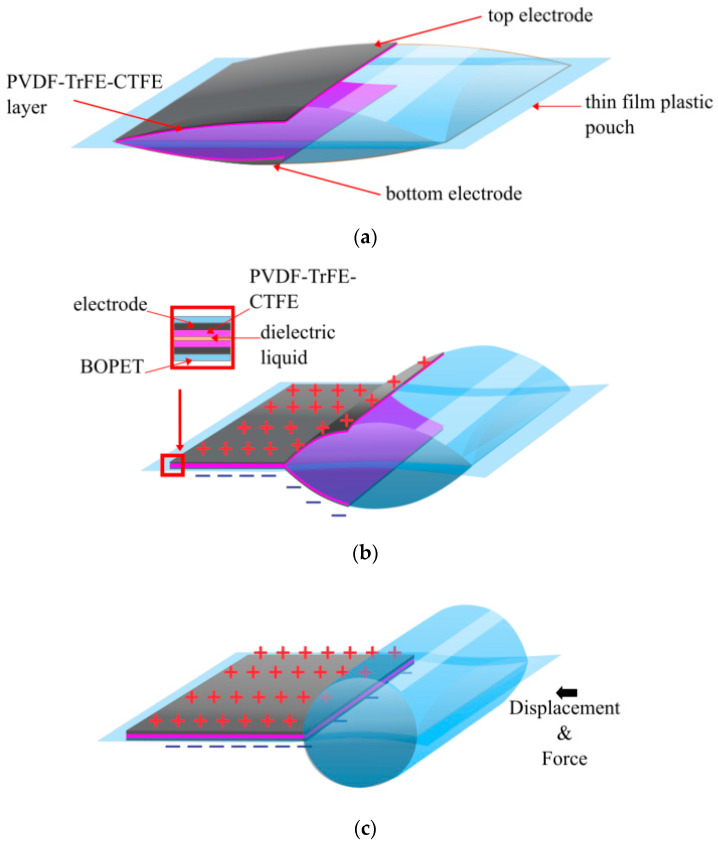
The HALVE actuator: (**a**) at rest; (**b**) when a voltage is applied, and the pouch is partially zipped; (**c**) when a voltage is applied, and the pouch is fully zipped, displaying the direction of the force and the displacement [18].

Reducing the gap between electrodes and increasing the permittivity massively reduces the voltage required to produce an electrostatic force. The Hydraulically Amplified Low-Voltage Electrostatic (HALVE) actuator Figure 5, was able to produce a free strain of ~9% and blocked up to 5 N (maintaining 2% strain) with an applied voltage of only 1.1 kV [18]. The maximum specific power was 50.5 W/kg. The design was essentially the same as the Peano HASEL actuator. However, it flipped the 12.5 µm BOPET pouch so that the electrodes faced each other, separating them with a 5 µm PVDF-TrFE-CTFE deposited on each electrode (Figure 5). Thus, rather than having the BOPET as the dielectric layer, it insulates the user from high voltages. Though the HALVE could operate up to 1.3 kV, voltage breakdown events (shorting between electrodes) were reported at voltages as low as 500 V due to impurities in casting the dielectric. Though the fluid dielectric layer allows the insulation layer to be restored, the dielectric layer is still damaged, degrading more and more with each breakdown. The voltage of 800 V was considered a reliable operating voltage.

Applications of the Peano HASEL include grippers [5,19,20], prosthetics [21,22], pumps [23], and locomotion [24,25,26,27,28,29,30]. Some grippers use the soft hydraulic pouch to grip objects [5,19], while others transfer the hydraulic force to a structure with hinges that bend [20]. This is distinct from the hinge actuators discussed below, as the electrodes are not integrated into the hinge. A unique application of the Peano HASEL actuator uses three actuators to displace a surface that objects can be lifted on and balanced [31]. Another unique design displaces this hydraulic force into an accordion-inspired design to produce angular motion [32,33], and another similar design called the Cutaneous Electrohydraulic (CUTE) is used in wearable haptic applications [34]. Research was also conducted by applying a secondary set of low-voltage electrodes, which provided a method of sensing the position of the Peano HASEL [35,36]. Biodegradable designs have also been researched to reduce the environmental impact of future designs [37].

### 2.3. Donut HASEL Actuators (Thin Film Plastic Actuator)

**Figure 6:**
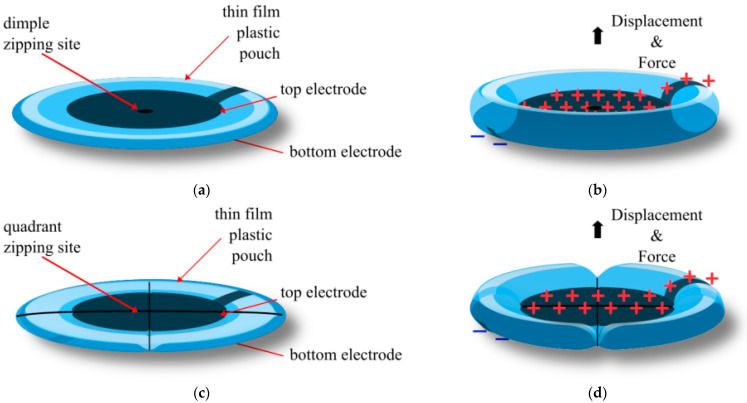
Donut HASEL actuator, with two types of zipping regions. (**a**) The dimple Donut HASEL actuator with only one zipping site at rest; (**b**) The dimple Donut HASEL actuator when a voltage is applied; (**c**) The Quadrant HASEL actuator with four lines of zipping sites; (**d**) The Quadrant HASEL actuator when a voltage is applied [38].

Unique fabrication methods have allowed the HASEL actuator to be more rapidly developed. This method of fabrication used a 3D printer nozzle to heat seal thin film soft pouches into any geometry required [38]. With this, the Donut Peano HASEL actuator was developed, as shown in Figure 6. Two types of Donut HASEL actuators were developed: the Dimple (Figure 6a) and the Quadrant Donut HASEL actuator (Figure 6b). The Dimple HASEL actuator sealed the centre of the pouch to create a single zipping site, much like the Peano HASEL actuator. On the other hand, the Quadrant HASEL actuator sealed the pouch into four quadrants, creating four zipping sites instead of one. This further reduced the voltage required for initial zipping and increased the initial force produced by the HASEL actuator.

The Quadrant HASEL actuator allowed for compact stacking of actuators, with a high transmission of force and displacement. The dimple HASEL actuator could produce a higher strain than the quadrant HASEL actuator, where with a 10 kV applied voltage and a 500 g load (4.905 N), the strains were 56% and 41%, respectively. However, when stacking actuators to increase the force and the displacement, the Dimple HASEL actuator had a significant reduction in strain compared to the Quadrant HASEL actuator, whereas with three stack actuators at 10 kV and a 500 g load (4.905 N), the strains were 20% and 72%, respectively. With three stacks for Quadrant HASEL actuators and an applied voltage of 12 kV, the free strain produced was 120% and a corresponding blocking force greater than 60 N. The maximum specific power was 121 W/kg, and an average specific power of above 60 W/kg at a load of 500 g. Unfortunately, there is a significant drop-off in efficiency as the actuators are stacked, requiring the segmenting of actuators with a rigid layer after a certain number of stacks [38].

Applications for the donut HASEL actuators include grippers [4] and appendages [38]. Other expanding HASEL actuator has a design similar to the donut, though instead of a revolved design, it is a linear design [39]; it is used for applications like a spring toy-inspired design [40], and it is even used as a needle biopsy robot and a rotary joint robot [41]. Similar quadrant designs have also been implemented into designs to induce rolling and twisting [42], as well as actuators that can perform locomotion [43].

### 2.4. Electrohydraulic Hinge Joints

**Figure 7:**
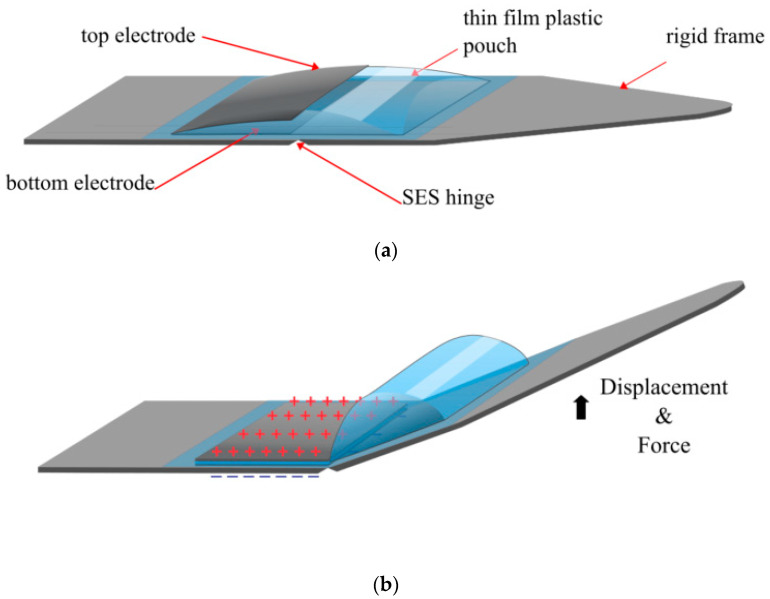
The SES joint: (**a**) when at rest, depicting the electrodes, rigid layers, thin film layers and the SES hinge; (**b**) when a voltage is applied [44].

Along with the quadrant actuators, this paper also demonstrated how HASEL actuators could bend, twist and curl by adding rigid layers, corrugated designs and spiralled patterns. Building on this work and inspired by the mechanics of spider legs, a new type of electrohydraulic actuator, referred to as a Spider-inspired Electrohydraulic Soft (SES) actuator, was developed [44,45]. Attaching a rigid lever arm to the pouch of the Peano actuator generated a large rotational force (Figure 7). The pouch was still made with BOPP, while acrylics were used to make the rigid material. As this is rotational movement, force metrics were replaced by a torque of 70 m-Nm, with a specific torque of 21 Nm/kg. The strain was replaced with degrees of rotation, which reached a maximum of approximately 70°. However, the power metrics are comparable to other HASEL technologies, with the maximum specific power being 230 W/kg. The specific energy was reported as 10.3 J/kg, which is quite high for the Peano HASEL actuator [5,12].

The hinge joint has been applied in most applications of all the electrohydraulic actuators. The most common of these applications is grippers [44,46,47]. Many of these grippers have multiple hinge stages for maximum rotation [20,46]. There are also many bio-inspired designs, including marine-inspired designs, such as the Mata stingray-inspired actuator [48] and a soft flipper electrohydraulic design [49] that moves through the water, and terrestrial-inspired designs, such as the eagle-inspired actuator mimics talons [46] and the spider-inspired actuator discuss above [44]. Even unique applications like shape-changing origami designs were able to be achieved [50,51], as well as designs enabling locomotion [52]. There is also research into developing biodegradable gripper designs along with other electrohydraulic actuators [53].

**Figure 8:**
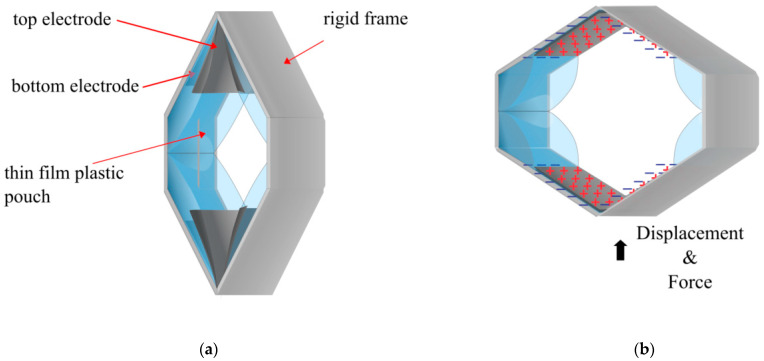
The HEXEL actuator: (**a**) when at rest, depicting the electrodes, rigid layers, and thin film layers; (**b**) when a voltage is applied [54].

Leveraging this energy gain, multiple SES joints were combined to develop the Hexagonal Electrohydraulic (HEXEL) actuator [54]. The HEXEL converted the rotational motion of the SES back into linear actuation (Figure 8). The rigid component used was fibreglass, though using magnetic tiles for the rigid component meant HEXEL actuators could be linked together to increase force or displacement. The HEXEL also switched the pouch material to 15–30 µm Polyethylene Terephthalate (PET). As the HEXEL expanded on one plane and contracted on the other, measurements were taken for each. A maximum free strain of 47.7% was reported when contracting, and a blocking force of 37.6 N. Notably, it was reported that at 1% strain, the maximum force was 12 N. The specific peak power and specific energy produced were 122 W/kg and 2.3 J/kg, respectively. When expanding, the strain was reported to be 113% with a blocking force of below 2 N and a maximum specific power of 90 W/kg.

### 2.5. Annular Electrohydraulic Actuator

Significant research into wearable haptics based on HASEL actuators has been carried out at EPFL over the past number of years.

**Figure 9:**
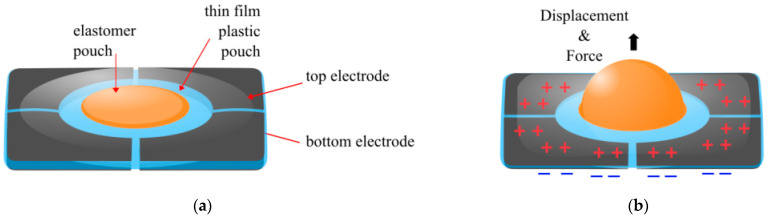
Schematic of HAXEL actuator combining stretchable, compliant, and rigid materials. (**a**) Actuator with no voltage applied, and (**b**) actuator with voltage applied [56].

Inverting the geometry of the Donut Peano HASEL [38] allows the fluid to be displaced through a central channel [55,56,57]. This annular electrode design was called the Hydraulic Amplified Taxel (HAXEL) actuator. The working principle and schematic of a HAXEL is shown in Figure 9. This name was given because the actuator was demonstrated as a wearable haptic device that produced a tactile force.

By dividing the electrodes into quadrants, HAXEL was also able to apply a shearing force that the user could perceive as a left, right, up, or down displacement, as well as a rotational displacement of clockwise or counter-clockwise. The new design and fabrication techniques combined compliant plastics (using 12 µm Mylar, DuPont, Wilmington, DE, USA), rigid plastics (using 100 µm PET) and elastomers (using 50 µm PDMS) to overcome the issues of previous elastomer designs. The PET and the Mylar had a metallised coating to create the electrodes, and the remaining metal was chemically etched. The resistive layer was deposited by a solid inkjet printer, making it quite easy to develop new and low-cost designs, adapted from previous work where inkjet printers were used to deposit carbon electrodes onto elastomers [58,59]. The maximum voltage applied to the HAXEL was 2 kV, which was the lowest maximum voltage so far; while this is safer for users in the long run, in the short term, it means that less electrostatic force can be produced compared to other designs. With a maximum voltage of 2 kV, the free strain reported was 60%, and a blocking force was 100–800 mN. The maximum specific power was reported to be 102 W/kg with a maximum peak specific energy of 0.51 J/kg.

Researchers at the University of Trento worked on electrohydraulic technology similar to HASEL actuators in 2017, a year before its development, with the Dielectric Fluid Transducers (DFT) [60,61]. The DFT used dielectric fluids between two elastomers, though this research mostly focused on electrostatic generators.

**Figure 10:**
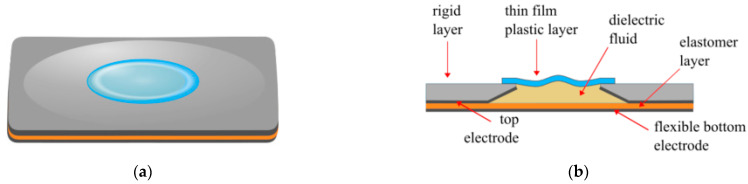

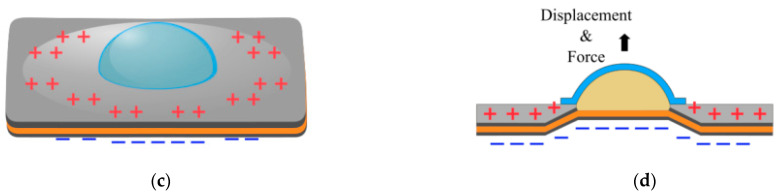
LEAP actuator: (**a**) when at rest; (**b**) a cross-sectional view of the actuator at rest; (**c**) when a voltage is applied; (**d**) a cross-sectional view of the actuator when a voltage is applied, demonstrating the direction of the force and displacement.

Building on the work with DFT, the Liquid-based Electro-Active Polymer transducer (LEAP) was developed [62]. LEAP technology was used to develop a haptic device, much like the HAXEL actuator, though there are a few notable differences (Figure 10). The elastomeric top layer was replaced with a compliant 10 µm PET, attached to a rigid acrylic frame. At the bottom of the acrylic frame is a conical indentation covered with 50 µm PDMS film. Though there are a few other layers in the experimental setup, this is essentially the layout. The compliant layer initially has a plastic deformation. When a voltage is applied, the elastomer is pulled toward the rigid frame, displacing the fluid up a centre channel and displacing the compliant layer. With an applied voltage of 4.5 kV, a 17 mN blocking force was generated.

### 2.6. Electro-Ribbon Actuator

Hydraulic principles of HASEL technology were reimagined entirely by the researchers at the University of Bristol. This work questioned whether the electrohydraulic actuator needs to displace a whole pouch of fluid [63,64,65,66]. While this removes the hydraulic force produced, it gives great insights into the fluid’s contribution to the electrostatic forces.

**Figure 11:**
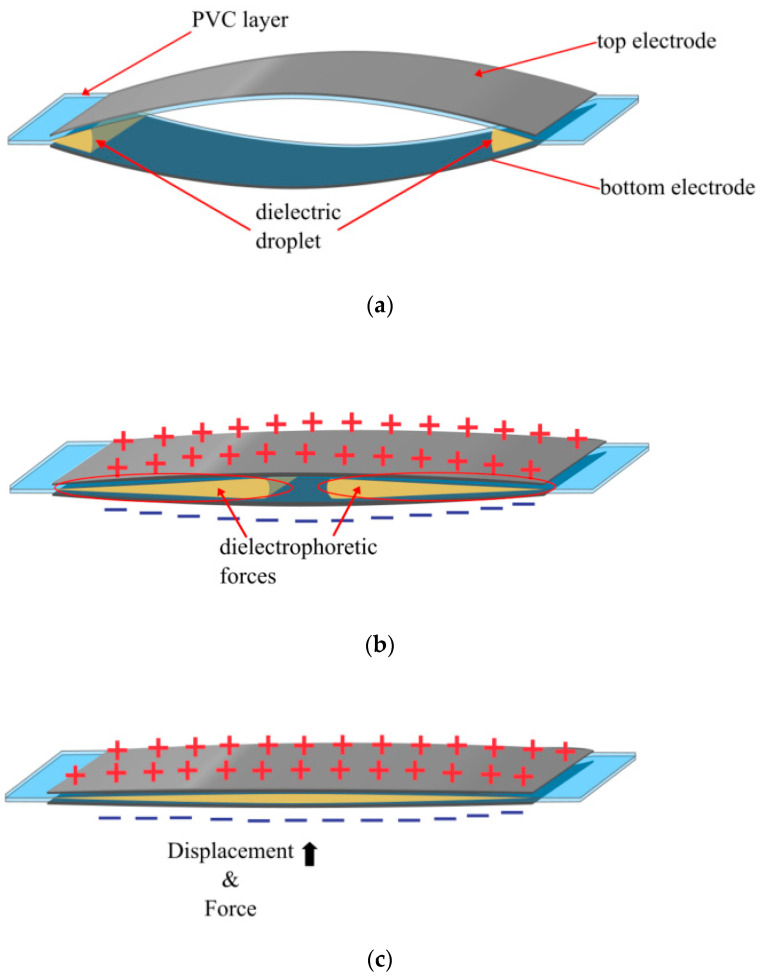
The Electro-ribbon actuator: (**a**) No voltage is applied, electrodes are bowed under the weight of the load, and the dielectric fluid is at the zipping region of the electrodes;(**b**) when a voltage is applied and the actuator is partially zipped, showing the fluid spreading along the insulator held by dielectrophoretic force; (**c**) when a voltage is applied, and the actuator is fully zipped, displaying the direction of the force and the displacement [65].

This work introduced a new type of electrostatic actuator called the Electro-ribbon actuator (Figure 11). The Electro-ribbon actuator eliminated the fluid pouch from the design entirely, replacing it with a single fluid droplet. The droplet is placed in the zipping region, as almost all the electrostatic force occurs here. Dielectrophoretic forces [67], a form of electrostatic force, keep the droplet stuck to the electrode. The electrodes were made of thin steel, insulated by 130 µm Polyvinyl Chloride (PVC) film, and silicone oil was used for the droplet. From 5 kV, the actuator was reported to have a free strain of greater than 99%. At 10 kV applied voltage, a load of 17.6 g (approximately 172.65 mN) is seen, though this is not necessarily the blocking force of the actuator [65]. More recent designs replaced the PVC film with Polyvinylidene fluoride-co-hexafluoropropylene (PVDF-HFP), improving the performance of the Electro-ribbon actuator [68].

### 2.7. Electrohydraulic Actuators with a Reservoir

**Figure 12:**
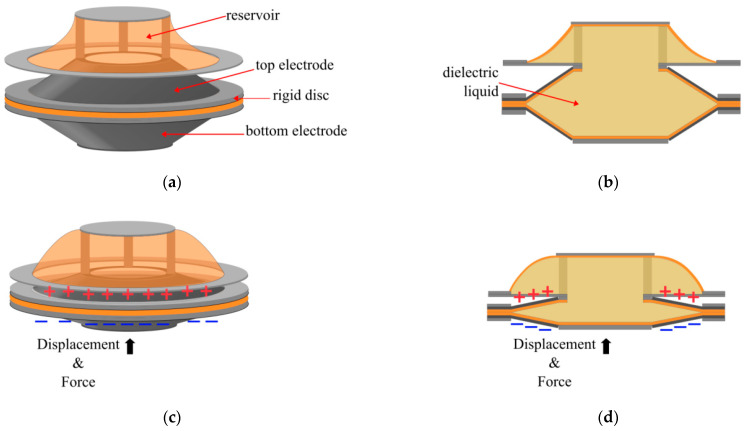
Electrostatic Bellow Muscle actuator (**a**) No voltage is applied, and the fluid is in the actuator (**b**) A cross-sectional view of the actuator when at rest (**c**) The actuator is fully zipped, and all of the fluid is now in the reservoir; (**d**) The cross-sectional view when a voltage is applied [69].

The Electrostatic Bellow Muscle (EBM) actuator was completely redesigned by adding a reservoir to the top of the actuator (Figure 12) [69]. The annular electrode design of the LEAP was maintained, but the whole pouch was now made with compliant 25 µm Polymide (PI) rather than combining rigid and compliant materials. A rigid annular ring was also used to keep the PI pouch together. The electrodes also had an annular configuration and were made of carbon paint. A hole in the centre of the PI pouch connected the pouch to the upper reservoir. The EBM was tested with one actuator unit up to six series stacked units. With an applied voltage of 8 kV, the six-stack EBM produced a free strain of 43%, and forces were tested up to approximately 7 N, though this was not necessarily the blocking force. The peak specific power for three series EBMs was 31 W/kg.

#### Dielectrophertic Actuator with a Reservoir

**Figure 13:**
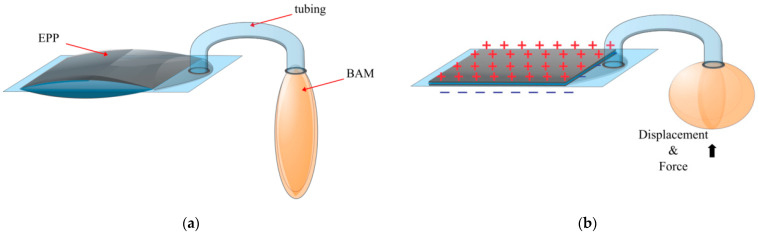
The Electro-Pneumatic Pump and BAM (**a**) at rest and (**b**) when a voltage is applied.

Using the Electro-ribbon actuator, a pneumatic pump was developed, referred to as the Electro-Pneumatic Pump (EPP) [64]. Much like the reservoir on the EBM, the EPP pumped fluid externally to displace a load (Figure 13). As it is based on the Electro-ribbon actuator, the displaced fluid is air. The pump was attached to a soft actuator developed in this work, called the Bubble Artificial Muscle (BAM) [70], making the EPP-BAM actuators. Force and displacement can be increased by increasing the initial pressure in the EPP. With applied voltages up to 10 kV, the EPP-BAM reportedly produced a maximum strain of 32.4%, with a maximum reported load of 100 g (0.981 N). The peak specific energy and specific power were reportedly 2.59 J/kg and 112.16 W/kg, respectively.

Besides the applications demonstrated by the EBM and the EPP-BAM, the most common application of the reservoir is the haptics for wearable devices [23,71,72].

## 3. Discussion

Analysing the electrohydraulic actuators requires a comparison of expanding and contracting actuators. This is due to the fact that expanding actuator movement (stretching) expands beyond the initial length of the actuator and does not have limitations. On the other hand, contracting actuators (squeezing) are constrained within the initial length of the actuator, limiting the strain to between 1% and 100%. Thus, these values do not correlate with performance when comparing strains. Overall strain is a good measurement for comparison as it is a normalised value.

Blocking force, while crucial for understanding an actuator’s absolute limit, is challenging to compare across devices, as it is not normalised. It is often not normalised in reporting from researchers either. This makes it less practical for real-world applications, where forces at 1–2% strain are often reported instead. Nevertheless, it provides insight into the maximum force levels explored in the field. Blocking force is a value that can be compared between expanding and contracting actuators.

Specific power is one of the best indicators of performance as it is a normalised value, and it correlates to performance in the same way between both expanding and contracting actuators.

The applied voltage required indicates the longevity of the design in the field. Designs that require higher voltage are going to be easier to implement into applications for commercialisation as high voltages are more difficult to manage safely and affordably [18,73].

The metrics used for this analysis draw from the most reported metrics in the field and may need to be expanded on in future work.

### 3.1. Expanding Electrohydraulic Actuators

Expanding Electrohydraulic actuator performances have varied greatly over the years. Table 1 displays all the results from the discussions on expanding electrohydraulic actuators. Though there have been many improvements over the years, it often comes at the expense of other metrics. Despite this, the quadrant HASEL actuator was the highest-performing expanding actuator across almost all categories. This includes the highest performing in terms of strain, blocking force, and specific power, which for the quadrant HASEL actuator was 118%, ~60 N, and 614 W/kg, respectively. The highest performing actuator in terms of voltage requirements was the HAXEL actuator with a voltage of 2 kV operating voltage.

The Elastomer Donut HASEL actuator has some of the lowest-performing metrics. The 40% to 50% strain was the lowest reported across the expanding actuators. Though it produces higher force than haptic technologies, like the HAXEL and the LEAP, it requires 5–10 times the applied voltage. This is potentially due to the materials’ thickness. The specific power and energy metrics were not reported. Though one of the lower-performing actuators, it has been a catalyst for the electrohydraulic actuators in this paper.

The Planar HASEL actuator had excellent performance metrics, though it may have limited applications. The planar HASEL actuator reported great performance metrics across the board. It was reported as the actuator with the highest specific power and energy. However, the actuator relied heavily on gravity to extend the load and the restoring force of the elastomer material rather than the electrostatic force itself.

The Quadrant HASEL actuator seems to have the most potential for scaling the force and strain of electrohydraulic actuators. Ignoring the Planar HASEL actuator, the quadrant HASEL actuator has the highest specific power and energy performance across the extending actuators. This high specific performance is reportedly maintained with several actuators stacked, though it dropped off quite rapidly. Researchers need to use rigid spacers for every few stacks to maintain high specific values. This scalability is demonstrated by the fact that three stacked quadrant actuators produced the highest force and strain of all the actuators.

The HAXEL actuator has great potential for electrohydraulic actuator applications. The force produced is quite low, though this is not necessarily an issue as it can be perceived quite consistently by users in haptic applications. The strain is also quite low, only faring better than the Elastomeric Donut HASEL actuator. This can potentially be due to the fact that while the elastomer dot is extending, the compliant geometry constraining the actuator is pulling the elastomer in the opposite direction. This could potentially be resolved by drawing from the LEAP design, where the dot is attached to a rigid structure that does not move relative to the displacement. On the other hand, the required voltage is the second lowest of all the electrohydraulic actuators used today, making it much easier to create a power supply to drive it [18]. Overall, being the smallest actuator design, it should theoretically have a higher specific energy. Instead, it has the lowest reported specific energy. This indicates that a lot of optimisation can be gained in the design of the HAXEL. This will be explored further in the dielectric material section below.

The LEAP and Expanding HEXEL actuators complement and provide a great platform for the other high-performing actuators. Much like the HAXEL actuator, the LEAP requires a much lower voltage than other applications. The low force of the actuator again seems to suffer from the thickness of the dielectric, as will be discussed below. Despite this, the LEAP created a great platform from which the EBM actuator could be developed. The HEXEL actuator is primarily a contracting actuator, of which the expanding metrics are mostly a complementary product. These values are quite low compared to the contracting actuator.

### 3.2. Contracting Electrohydraulic Actuators

Contracting electrohydraulic actuators receive much more attention in the field than expanding actuators. This is because contracting electrohydraulic actuators resemble muscle-like movement. Table 2 displays all the results from the discussions on contracting electrohydraulic actuators. Much like the expanding actuators, the contracting actuators seem to struggle to have inconsistent performances in their metrics from design to design. The Electro-ribbon actuator achieved the highest-performing strain with a strain of 99%. The Peano HASEL actuator had the highest blocking force at 60 N. The SES joint had the highest specific power at 230 W/kg. The HALVE actuator had the best voltage performance at 1.1 kV.

Despite being one of the first HASEL designs, the Peano HASEL actuator produced some of the highest scores in many categories. The Peano HASEL actuator has gone through the most optimisation of any designs discussed in this paper. This has allowed it to produce the highest force of all the contracting actuators. However, it also produces small strains across the design. As discussed above, having such low displacement means very few applications can use this design, as very little work is completed. This is primarily due to the electrode length taking up most of the pouch length. The HS Peano HASEL actuator was developed specifically to combat this issue. The specific power and energy of the Peano actuator are relatively good compared to other both contracting and expanding actuators.

The HS Peano HASEL actuators improve on some metrics of the Peano HASEL while decreasing the performance of others. Though the strain has increased for the HS Peano actuator, it is not even close to the highest-performing strain for a contracting actuator. This low gain in strain also produces a large decrease in the blocking force of the HS Peano. The low strain voltages of these designs are due to the fact that when hydraulically deforming a soft material, the minimum geometry the pouch can be deformed into is a sphere [74]. This means that if high strains are to be achieved, the force applied to the load needs to be directly driven by the electrostatic force of the actuator. The exception to this is the HEXEL actuator, which is discussed below. The HS Peano actuator, however, maintains the specific power and energies produced by the Peano actuator.

Work on the SES joint provided a new direction for Peano HASEL research. Adding rotational force to the Peano HASEL actuator greatly improved many of the metrics of the previous designs. Though no force and strain metrics can be produced, the SES joint produced the highest specific power and energy metrics of any electrohydraulic actuator, excluding the planar HASEL actuator.

The HEXEL actuator drastically improves the high strain of the Peano HASEL actuator while maintaining a high force. Using the SES joint allowed the HEXEL to overcome the low strain of the Peano and the HS Peano actuator while still being driven by a hydraulic force. Unfortunately, the high specific power and energy of the SES joint were not present in the HEXEL metrics. This suggests significant losses as more SES joints are added. Further work on reducing these losses and optimising the design will hopefully regain much of that energy loss. The HEXEL actuator creates a great trajectory for the researchers at Boulder University.

The EBM and EPP–BAM actuator’s use of the reservoir introduces new advancements to the field but creates new challenges. The first challenge is the fact that the EBM has to expend energy displacing the reservoir, which could be used to lift the load. This potentially explains its low power output. Also, though the EBM produces high strain, the location and storage of the reservoir must be considered in applications. These issues seem to become more complicated, requiring more than one actuator. Do you use multiple reservoirs? Do you need to use one reservoir and create situations where one actuator would have to displace a reservoir built for ten? Having the EPP displace another soft device, like the BAM, is a good solution, as the EBM was also demonstrated to work as a pump. However, this takes away one of the key benefits of electrohydraulic actuators because they can produce force locally. Adding reservoirs and tubing reintroduces failure points into the design.

The Electro-ribbon actuator almost reaches the theoretical limit of the contracting electrohydraulic actuator strain. The Electro-ribbon actuator was a great example of taking the concept back to the first principal’s question of how much dielectric fluid is really required. However, the force produced by the actuator is very low, and it is difficult to compare the Electro-ribbon actuator to other electrohydraulic actuators without specific power or energy metrics, although it seems that a few design adjustments could easily improve the power produced by this actuator.

The HALVE actuator demonstrates how much the performance can be improved with the electrohydraulic actuator performance by improving material selection. With the lowest voltage used in any of the actuators, the HALVE was able to surpass the performance of most of the actuators in this paper. Concepts from this work could be implemented into all the electrohydraulic actuators discussed in this work to improve performance. These concepts will be discussed below. The low breakdown voltage of the HALVE will be a sign.

### 3.3. Electrostatic Force Analysis

The most common force analysis method for electrohydraulic actuators is analysing the electrostatic force between two parallel plates. The parallel plate analysis is a steady-state analysis in which a voltage has been applied, and the pouch has been fully zipped. Thus, as the dielectric fluid has been squeezed in this state, it can be ignored, and only the dielectric pouch material remains between the electrodes. Collecting key dielectric properties of the dielectric pouch and comparing them with an electrostatic force analysis gives insight into the performance of each electrohydraulic design.

The formula for calculating electrostatic force between parallel electrodes is as follows:

(1) $$F=\frac{\varepsilon_{r}\times \varepsilon_{o}\times A{\times V}^{2}}{2\times d^{2}},$$

where *ε_r_* is the relative permittivity; *ε_o_* is the permittivity of free space; *A* is the overlapping electrode area; *V* is the applied voltage, and *d* is the gap between the electrodes. The variables related to the dielectric materials in the steady-state analysis are the relative permittivity and the gap between the electrodes. The pouch material determines the relative permittivity, and the electrode gap is determined by the pouch’s thickness and the number of pouch layers between the electrodes.

Table 3 collects the dielectric pouch materials implemented in each electrohydraulic actuator. Data have been collected from values reported in their respective publications. The values included in this table are the materials used, the thickness of the material used, the number of pouch layers between the electrodes, and the resultant pouch thickness. Relative permittivity reported by each publication was also included.

### 3.4. Dielectric Pouch Materials

Electrohydraulic Actuators have used many pouch materials over the years. The dielectric permittivity is very wide-ranging. From Equation (1), it can be observed that the relationship between material permittivity and electrostatic force is linear. The blue lines plot the trajectory of the force due to the relative permittivity.

The first materials were elastomeric materials with PDMS. Though this is a common selection for DEAs, it is not a very common selection for electrohydraulic actuators. This is potentially due to the relatively average permittivity values [75]. The fabrication method for PDMS generally requires mixing and casting for each pouch. This adds to the fabrication time for researchers and makes it difficult to ensure the consistency of each layer.

The most common material used for the pouch was BOPP, as it was the main material used in Peano actuators. BOPP is polypropylene (PP) stretched in two directions during fabrication to increase strength and durability [76]. However, BOPP generally has fairly low permittivity, reducing the electrostatic force that can be produced through it [77,78]. The stiffness of the BOPP also restricted some of the designs, such as the HS Peano HASEL actuator, though this may have been more of an issue with the design of the actuator. TPU was used to overcome the stiffness issue, and it was reported to have quite high permittivity values. Unfortunately, TPU has a much lower breakdown voltage. The permittivity of the material also varies a lot, and the permittivity also reduces the hardness [79].

Instead of using BOPP, as used in much of Boulder University’s work, EPFL used Mylar, which is a Biaxially Oriented Polyethylene Terephthalate (BOPET), which is PET that, like BOPP, has been stretched in two directions during manufacturing. Mylar is a material commonly used in electronics for its dielectric properties, which are commonly higher than BOPP [80,81]. Other advantages of PET are that it has a much higher melting temperature and is more UV resistant than PP [82]. One of the downsides of PET is that it absorbs much more moisture than PP, affecting permittivity performances in different temperatures [83]. It also has a lower breakdown voltage, increasing the chance of voltage breakdown. For the HEXEL actuator, researchers at Boulder University even switched to using BOPET.

The other thin film plastic used was PI, with the EBM actuator. PI generally has a similar relativity-making [84]. It can also handle higher temperatures and has a higher breakdown voltage compared to PET [77].

PVC was another material used with the Electro-ribbon actuator, which had properties similar to those of the other polymers. PVC is an excellent insulator used in many applications and exhibits many similar qualities to other materials [85].

The most interesting material that could be implemented into many future designs is PVDF-TrFE-CTFE [86]. This material has recently started to be used in electrohydraulic actuators with the EPP and the HALVE actuator. It reportedly has a relative permittivity of 40, about five times the permittivity of any other material used so far. One issue with fabrication with this material is that it has been difficult to deposit the material onto surfaces, such as with the HALVE actuator, increasing the chance of dielectric breakdown. Mitigating the risk of breakdown events in future designs requires the fabrication of a uniform PVDF-TrFE-CTFE without defects. The other way to mitigate this breakdown issue is to reduce the required voltage for future designs.

### 3.5. Dielectric Pouch Thickness

**Figure 14:**
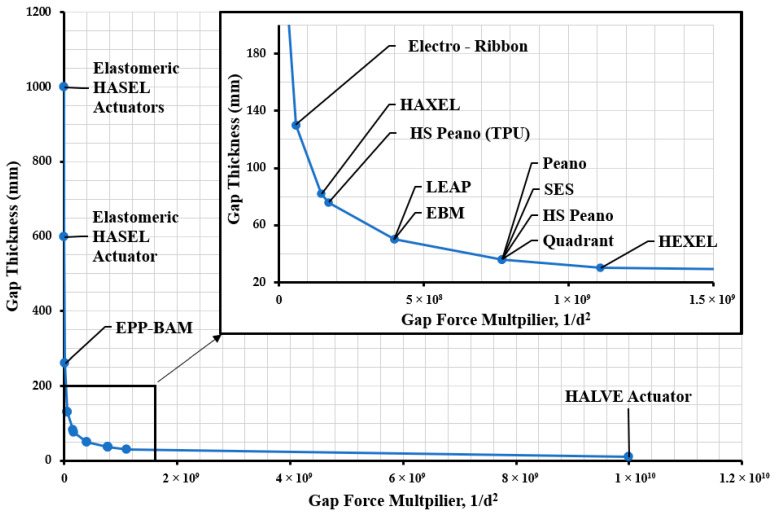
Graph comparing the electrode gap of the various electrohydraulic actuators used for the dielectric pouches over the years and their relationship to force production.

The thickness has a massive multiplier effect on the electrostatic force of the electrohydraulic actuator. From Equation (1), we can see that the electrode gap has an inverse square relationship with electrostatic force. Using the values from Table 3, force multiplication values were determined. The comparison of each actuator force multiplication due to the electrostatic gap is shown in Figure 14. The blue lines plot the trajectory of the force due to the gap.

Elastic materials are generally restricted in how thin they can be. This is because an elastomer generally needs to contract on the transverse plane when it is stretched. Hence, the largest gaps are exhibited by the elastomers like PDMS, and Ecoflex is used with the Elastomeric HASEL actuator, from 300 µm to 500 µm, respectively. This intern means the lowest force multiplication. The LEAP was able to achieve a resolution of 50 µm, which was quite a significant improvement. The HS Peano HASEL actuator also had a large gap when using the TPU.

The Researchers at Bristol design used dielectric layers that were quite thick compared to other inelastic designs. For both the Electro-ribbon actuators and EPP, the dielectric material was 130 µm. The Electro-ribbon actuator significantly increased the force of the EPP as it only required one layer between the two electrodes.

From the LEAP and EBM onwards, there is essentially an inflexion point for force production. From this point, the force produced significantly increases with each decrease in the electrode gap.

University of Colorado Boulder researchers have used many of the same materials throughout the years, which helps them focus on the overall design of their electrohydraulic actuator. This can be attributed to the fact that they are already working with significantly thinner materials than many other researchers in the field. The use of the thinner PET material with HEXEL, reducing the gap from 36 to 30 µm, increased the electrostatic gap from 7.72 × 10^8^ to 1.11 × 10^9^.

The largest gap force multiplier was again calculated from the HALVE actuator. With a gap of only 10 µm, the force produced was 1.00 × 1010, which was drastically larger than any of the previous calculations. Again, it is important to note that there is also an increase in dielectric breakdown events at this size. Fabrication methods need to be improved to make this actuator more reliable.

### 3.6. Electrostatic Force in Electrohydraulic Actuators

It has been demonstrated previously that force multiplication reduces electrode gap [74]. The exponential increase in force becomes apparent as the electrode gap becomes smaller. However, the biggest gains are seen when both permittivity and electrode gap are leveraged to achieve the maximum force multiplication.

**Figure 15:**
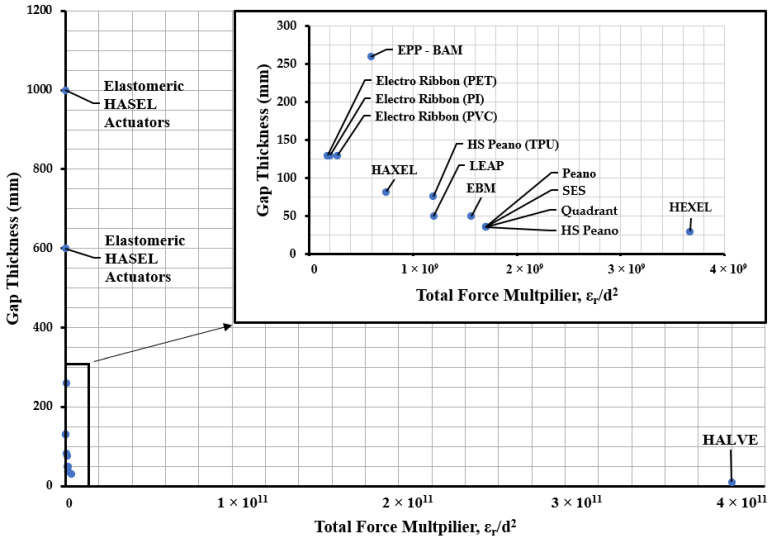
Graph comparing the electrode gap with the relative permittivity of the various electrohydraulic actuators used for the dielectric pouches over the years and their relationship to force production.

Figure 15 demonstrates the total electrostatic force contributed by the dielectric pouch material. None of the positions have really changed. However, the magnitude has significantly changed. The force multiplier produced by the HALVE actuator was 4.00 × 10^11,^ with the next closest being the HEXEL actuator with 3.67 × 10^9^.

## 4. Conclusions

In recent years, electrohydraulic actuators have undergone many advancements. Many new designs for expanding and contracting actuators have been developed, expanding principles in the field and improving performance. The Quadrant Donut HASEL actuator had the highest overall performance for the expanding actuators, while the HEXEL actuator had the greatest average performance for the contracting actuators. Overall, the Quadrant Donut HASEL actuator performed better in most areas than the HEXEL actuator. The HALVE actuator was able to achieve high-performance metrics while maintaining a much lower voltage than other applications. This shows great promise for the field going forward.

Performance metrics need to be improved before electrohydraulic actuators can be widely adopted. Though many principles have been added to advance the field, future work needs to focus on optimising the performance of designs. Work like the Electro-ribbon actuator has greatly contributed to challenging principles in the field with the dielectric droplet. However, the low force affects its viability for applications, which is the largest gap in the field, along with low specific power performance and low strain overall. Implementing smaller gaps between the electrodes and using materials with higher dielectric properties was shown to improve the performance and safety of actuators. However, these smaller gaps increase the likelihood for the actuator to break. Future actuator designs should focus on optimising performance metrics.

## Figures and Tables

**Table 1:** Expanding Electrohydraulic actuator metrics as reported over the years. Results from each university are displayed together. Only steady-state values are reported in the table. The table did not consider metrics reported from oscillating input voltages, like the Planar HASEL actuator.

Actuator	University	Applied Voltage (kV)	Max. Free Strain (%)	Max. Blocking Force (N)	Peak Specific Power (W/kg)	Peak Average Specific Power (W/kg)	Specific Energy (J/Kg)
Elastomeric Donut HASEL actuator	University of Colorado Boulder	21	40–50	2.45–3.92			
Planar HASEL actuator	University of Colorado Boulder	~22.5	79	2.45–14.72	614		70
Three-stack Quadrant HASEL actuator	University of Colorado Boulder	12	118	~60 *	121	>60	12
HEXEL	University of Colorado Boulder	9.5	113	>2	90	~30	
HAXEL	EPFL	2	60	0.1–0.8	102		0.51
LEAP	University of Trento	4.5		0.017			

* The heat seal broke at 60 N.

**Table 2:** Contracting electrohydraulic actuator metrics, as reported over the years. Results from each university are displayed together.

Actuator	Institution	Applied Voltage (kV)	Max. Free Strain (%)	Max. Blocking Force (N)	Peak Specific Power (W/kg)	Peak Average Specific Power (W/kg)	Specific Energy (J/Kg)
Three-stack Peano HASEL actuator	University of Colorado Boulder	10	9–15	9.81–60	160	>50	4.93
HS Peano HASEL actuator	University of Colorado Boulder	10	24	18	~120	~78	4.03
SES	University of Colorado Boulder	9			230	110	10.3
HEXEL	University of Colorado Boulder	9.5	47.7	37.6	122		2.3
Three–Six series EBM	University of Trento	8	43	~7 *	31		
Electro-ribbon Actuator	University of Bristol	10	>99	0.172 *			
EPP-BAM	University of Bristol	10	32.4	0.981 *	112.16		2.59
HALVE actuator	ETH Zürich	1.1	9	5 *	50.5		

* This was the maximum force reported, not necessarily the blocking force.

**Table 3:** Displays all the dielectric pouch materials used in the electrohydraulic research discussed in this paper.

Actuator	Institution	Dielectric Material	Dielectric Thickness (µm)	Dielectric Layers	Total Dielectric Gap (µm)	Relative Permittivity	Compliant/Elastomeric
Elastomeric Donut HASEL Actuator	University of Colorado Boulder	Ecoflex	500	2	1000	2.3–3	Elastomeric
		PDMS	300	2	600	2.3–3	Elastomeric
Elastomeric Donut HASEL Actuator	University of Colorado Boulder	Ecoflex	500	2	1000	2.3–3	Elastomeric
		PDMS	300	2	600	2.3–3	Elastomeric
Peano HASEL actuator	University of Colorado Boulder	BOPP	18–21	2	42	2.2	Compliant
HS Peano HASEL Actuator	University of Colorado Boulder	BOPP	18	2	36	2.2	Compliant
		TPU	38	2	76	6.9	Elastomeric
Quadrant HASEL Actuator	University of Colorado Boulder	BOPP	18	2	36	2.2	Compliant
SES	University of Colorado Boulder	BOPP	18	2	36	2.2	Compliant
HEXEL	University of Colorado Boulder	PET	15–30	2	30–60	3.3	Compliant
HAXEL	EPFL	PET	12 50–100 *	1 1	82–132	3.3	Compliant
		PVDF-TrFE-CTFE,	5 15 *	1 1		38	Compliant
LEAP	University of Trento	PDMS	50	1	50	2.3–3	Elastomeric
EBM	University of Trento	PI	25	2	50	3.9	Compliant
Electro-ribbon Actuator	University of Bristol	PET	130	1	130	3–3.4	Compliant
		PI	130	1	130	3.4–3.5	Compliant
		PVC	130	1	130	4.62	Compliant
EPP-BAM	University of Bristol	PVDF-TrFE-CTFE	130	2	260	40	Compliant
HALVE Actuator	ETH Zürich	PVDF-TrFE-CTFE	5	2	10	40	Compliant

* Bottom layer dielectric.

## Data Availability

All relevant data are included in this paper.
